# Association of MTHFR missense variants with thromboembolic diseases and coagulation factor levels in European populations

**DOI:** 10.1186/s12959-025-00711-1

**Published:** 2025-04-08

**Authors:** Iyas Daghlas, Mengmeng Wang, Dipender Gill

**Affiliations:** 1 Department of Neurology, UCSF Weill Institute for Neurosciences, University of California San Francisco, San Francisco, CA USA; 2 Department of Neurology, The Third Affiliated Hospital of Soochow University, Changzhou, China; 3 Department of Epidemiology and Biostatistics, School of Public Health, Imperial College London, London, UK

**Keywords:** Cardioembolic stroke, MTHFR, Proteomics, UK biobank, Venous thromboembolism

## Abstract

**Background:**

Investigations of the association between missense variants in the methylenetetrahydrofolate reductase (*MTHFR*) gene and thromboembolic diseases have been limited by small sample sizes. The effect of these variants on coagulation factor levels remains similarly uncertain.

**Objectives:**

To test the association of the C677T and A1298C missense variants in *MTHFR* with risk of venous thromboembolism (VTE), cardioembolic stroke (CES), and circulating coagulation cascade protein levels.

**Patients/Methods:**

We analyzed genetic associations of *MTHFR* missense variants with VTE (81,190 cases and 1,419,671 controls), CES (10,804 cases and 1,234,808 controls), and circulating levels of coagulation cascade proteins from the deCODE (*n* = 35,559) and UK Biobank (*n* = 46,218) cohorts. All participants in these genetic analyses were of European ancestry. We report odds ratios (OR) and beta coefficients per copy of the missense variant. VTE associations were compared to the effect of the Factor V Leiden variant.

**Results:**

The A1298C variant conferred a small increased risk of VTE (OR per allele: 1.03, 95% confidence interval [CI] 1.02–1.04, *P* = 1.36 × 10^− 6^). This effect was 30-fold weaker than the effect of Factor V Leiden on VTE. After correction for multiple comparisons, the C677T variant did not demonstrate a significant association with VTE (OR 0.99, 95% CI 0.98-1.00, *P* = 0.04). Neither variant was associated with CES (*P ≥* 0.18), nor with any of the 34 coagulation cascade proteins after correction for multiple comparisons.

**Conclusions:**

These data do not support a role for MTHFR genetic testing as part of an inherited thrombophilia evaluation.

## Introduction

Methylenetetrahydrofolate reductase (MTHFR) plays a key role in folate and homocysteine metabolism [1]. Common missense variants in *MTHFR* that reduce enzymatic function, including C677T and A1298C, have been investigated for associations with a wide range of clinical outcomes [1]. Evidence for their relationship with thromboembolic disease remains conflicting, with some studies suggesting increased risk [2–4], while others report no association [5, 6]. Inference from this body of literature is limited by inconsistent findings and relatively small sample sizes [2]. While the C677T variant has been extensively studied, research on the A1298C variant remains limited. Furthermore, there has been no systematic investigation of the association between these variants and levels of circulating coagulation cascade proteins.

To address these knowledge gaps, we conducted the largest investigation to-date of the association between the common *MTHFR* C677T and A1298C missense variants and risk of venous and arterial thromboembolism. To provide mechanistic insights, we further examined associations of these missense variants with levels of circulating coagulation cascade proteins. These variants were selected because they represent the most frequently occurring missense variants in *MTHFR*, are commonly included on clinical genetics panels, and have been the primary focus of research examining associations between *MTHFR* and clinical outcomes [7, 8].

## Methods

### Genetic associations with venous thromboembolism and cardioembolic stroke

Genetic associations with venous thromboembolism (VTE) were obtained from the largest available genome-wide association study (GWAS) meta-analysis comprising 81,190 cases and 1,419,671 controls from seven cohorts: deCODE, Copenhagen Hospital Biobank Cardiovascular Disease Cohort, The Danish Blood Donor Study, Intermountain Healthcare, UK Biobank, FinnGen, and the Million Veterans Program [9]. All participants were of European ancestry. Cases of VTE were identified primarily using International Classification of Diseases (ICD) codes for deep vein thrombosis or pulmonary embolism in hospital records, with additional self-reported cases from the UK Biobank cohort. Study participants across contributing cohorts were born between 1949 and 1962, with males comprising 45–52% of subjects and mean body mass index ranging from 27 to 29 kg/m^2^. All genetic associations were adjusted for patient age, sex, and genetic ancestry markers.

We included cardioembolic stroke (CES) as a phenotype of arterial thromboembolism. Genetic associations with CES were obtained from the GIGASTROKE GWAS meta-analysis of 10,804 CES cases and 1,234,808 controls of European ancestry [10]. Diagnosis of CES was based on the Trial of ORG 10,172 in Acute Stroke Treatment (TOAST) criteria [11]. The proportion of male participants in contributing cohorts ranged from 34 to 61%, and the mean age ranged from 42 to 83 years. All genetic associations were adjusted for age, sex, study-specific covariates, and genetic ancestry.

### Genetic associations with circulating levels of coagulation cascade proteins

A list of coagulation cascade proteins was generated by referencing the canonical coagulation cascade pathway identified in the Kyoto Encyclopedia of Genes and Genomes (KEGG) knowledge base (Entry hsa04610) [12]. These proteins spanned the intrinsic (e.g., coagulation factor XI and XII), extrinsic (e.g., coagulation factor VII), and common (e.g., fibrinogen, thrombin) pathways of coagulation. We obtained genetic associations with circulating levels of these proteins from the deCODE and UK Biobank (UKB) proteogenomic cohorts [13, 14]. The deCODE cohort included 35,559 Icelanders and measured circulating protein abundance using the aptamer-based SomaScan version 4 platform [13]. The mean age of participants was 56 years and 43% of participants were male. The SomaScan aptamer assay estimates relative protein levels in fluorescence units, and these were standardized through inverse rank normal transformation and adjusted for age, sex, and sample age. Genetic associations were estimated using a linear mixed model algorithm that controls for participant relatedness [15].

The UKB proteogenomic cohort measured circulating protein abundance using the antibody-based Olink Explore 3072 platform [13]. We used genetic associations that were estimated within the subset of 46,218 randomly selected participants of European ancestry. The mean age of participants was 58 years and 46% were male. Protein measurements underwent inverse rank normal transformation with adjustment for age, sex, and sample age. A linear mixed model was used to estimate genetic associations while adjusting for participant relatedness.

### Statistical analysis

We extracted genetic associations of *MTHFR* variants C677T (rs1801133) and A1298C (rs1801131) with VTE, CES, and all measured coagulation cascade proteins in the deCODE and UKB cohorts. We report odds ratios (OR) for disease outcomes and beta coefficients for protein levels, alongside 95% confidence intervals. Effect estimates were oriented to the missense allele that decreases MTHFR enzymatic activity [1]. To assess clinical relevance, we compared the VTE effect sizes of the *MTHFR* variants to the effect of Factor V Leiden (rs6025), the most prevalent inherited thrombophilia in European populations. This variant is routinely included in clinical thrombophilia screening panels, as its effect on VTE risk is considered sufficient to alter clinical management [16]. We report the ratio of the effect estimates of the two variants on the log-odds scale.

Statistical significance thresholds were adjusted for multiple comparisons. For analysis of clinical outcomes, we used a cutoff of *P* < 0.05/4 = 0.0125 to account for testing two outcomes across two variants. For analysis of protein levels, we used a cutoff of *P* < 0.05/2 × 34 = 7.35 × 10^− 4^ to account for testing associations of two variants with 34 protein levels; duplicate protein measurements across assays were not considered independent tests. All analyses were conducted using R version 4.2.0.

### Ethical approval

All contributing GWAS obtained informed consent from study participants. The present analysis was exempt from IRB approval due to the use of de-identified, summary-level genetic association data. Human Ethics and Consent to Participate declarations: not applicable.

### Data availability

All data used to support the findings in this manuscript are publicly available through links provided in the referenced manuscripts.

## Results

**Fig. 1:**
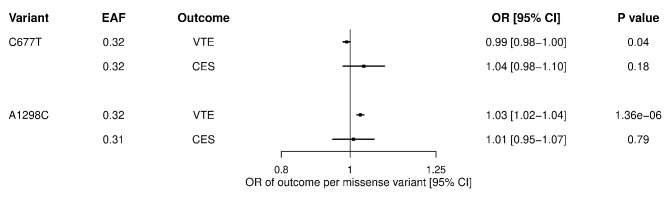
Associations of MTHFR missense variants with risk of venous thromboembolism (VTE) and cardioembolic stroke (CES). Forest plots display the point estimates (squares) and 95% confidence intervals (horizontal lines) for genetic associations. CI: confidence interval; EAF: effect allele frequency; OR: odds ratio

Genetic associations of the *MTHFR* missense variants with VTE and CES are shown in Fig. 1. After adjustment for multiple comparisons, the C677T variant was not associated with VTE (OR per effect allele 0.99, 95% confidence interval [CI] 0.98-1.00, *P* = 0.04), nor with CES (OR 1.04, 95% CI 0.98–1.10, *P* = 0.18). The A1298C variant showed a significant association with increased VTE risk (OR 1.03, 95% CI 1.02–1.04, *P* = 1.36 × 10^− 6^), but not with CES (OR 1.01, 95% CI 0.95–1.07, *P* = 0.79). For context, the OR for association of the Factor V Leiden variant (allele frequency 0.03) with VTE was 2.28 (95% CI 2.23–2.34, *P* = 2.17 × 10^− 916^). This represents a 30-fold stronger association relative to the A1298C-VTE association.

**Fig. 2:**
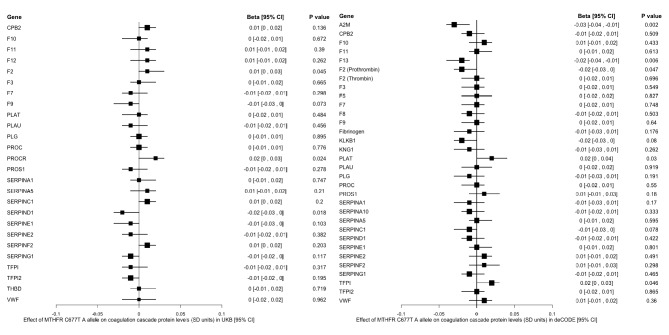
Associations of MTHFR C677T variant with levels of circulating coagulation cascade proteins in deCODE and UK Biobank (UKB). Forest plots display the point estimates in standard deviation units (squares) and 95% confidence intervals (horizontal lines) for genetic associations. With the exception of fibrinogen (encoded by multiple genes), proteins were labeled using corresponding gene names. CI: confidence interval; OR: odds ratio; SD: standard deviation

**Fig. 3:**
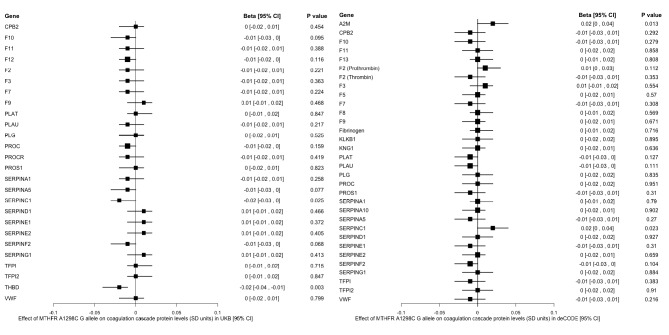
Associations of MTHFR A1298C variant with levels of circulating coagulation cascade proteins in deCODE and UK Biobank (UKB). Forest plots display the point estimates in standard deviation units (squares) and 95% confidence intervals (horizontal lines) for genetic associations. With the exception of fibrinogen (encoded by multiple genes), proteins were labeled using corresponding gene names. CI: confidence interval; OR: odds ratio; SD: standard deviation

A total of 34 unique coagulation cascade proteins were available for analysis across the deCODE and UKB datasets (listed in Figs. 2 and 3). After adjustment for multiple comparisons, there were no statistically significant associations of the *MTHFR* missense variants with levels of circulating coagulation cascade proteins. The strongest nominal association of the C677T variant was with reduced levels of alpha-2-macroglobulin (*P* = 0.002), and the strongest nominal association of the A1298C variant was with reduced levels of thrombomodulin (*P* = 0.003).

## Discussion

In this large-scale genetic analysis, we found no evidence for a clinically meaningful effect of *MTHFR* missense variants on risk of thromboembolic disease and on coagulation factor levels. Although A1298C showed a statistically significant association with VTE, the effect size was small, constituting a 30-fold weaker effect relative to the Factor V Leiden mutation. If this association were mediated by MTHFR perturbation, the C677T variant, which more strongly reduces MTHFR activity, would be expected to show a stronger link to VTE [17, 18]. However, the null effect of C677T on VTE suggests that the A1298C-VTE association may in fact result from genetic confounding or pleiotropic effects on nearby genes. Neither variant significantly influenced coagulation cascade protein levels. The associations of C677T with alpha-2 macroglobulin levels and of A1298C with thrombomodulin levels were only nominally significant and therefore require independent validation. Taken together, our findings support clinical guidelines that recommend against checking for *MTHFR* mutations in patients presenting with VTE [19].

Key strengths of our analysis include the large sample size, examination of both the A1298C and C677T variants, assessment of both venous and arterial thromboembolism, and investigation of proteomic outcomes. Importantly, all genetic associations were adjusted for genetic ancestry, addressing a methodological limitation of previous studies [2]. Several limitations warrant consideration. First, this study was restricted to individuals of European ancestry, limiting generalizability to other populations. Second, these studies were not stratified by folic acid status, leaving potential gene-environment interactions unexplored. Third, we did not have access to a VTE GWAS stratified by age to determine whether *MTHFR* variation influences VTE in younger versus older populations. VTE case identification relied primarily on ICD codes which may have introduced misclassification [20]; if random, such misclassification would generally bias genetic associations toward the null. Finally, we were limited to testing additive genetic models and cannot exclude other patterns of genetic association.

## Conclusion

These data do not support a clinically relevant association of missense variation in *MTHFR* with hypercoagulability in individuals of European ancestry. These findings support clinical guidelines that do not recommend *MTHFR* genetic testing as part of an inherited thrombophilia evaluation.

## Data Availability

No datasets were generated or analysed during the current study.

## References

[1] Schwahn B, Rozen R. Polymorphisms in the methylenetetrahydrofolate reductase gene. Am J PharmacoGenomics [Internet]. 2001;1:189–201. Available from: 10.2165/00129785-200101030-00004 10.2165/00129785-200101030-00004 12083967

[2] Den Heijer M, Lewington S, Clarke R, Homocysteine. MTHFR and risk of venous thrombosis: a meta-analysis of published epidemiological studies. J Thromb Haemost [Internet]. 2005;3:292–9. Available from: http://www.ncbi.nlm.nih.gov/pubmed/15670035 10.1111/j.1538-7836.2005.01141.x 15670035

[3] Liu F, Silva D, Malone MV, Seetharaman K. MTHFR A1298C and C677T polymorphisms are associated with increased risk of venous thromboembolism: a retrospective chart review study. Acta Haematol. 2017;138:208–15. 29212064 10.1159/000480447

[4] Ray JG, Shmorgun D, Chan WS. Common C677T Polymorphism of the methylenetetrahydrofolate reductase gene and the risk of venous thromboembolism: meta-analysis of 31 studies. Pathophysiol Haemost Thromb [Internet]. 2002;32:51–8. Available from: 10.1159/000065076 10.1159/000065076 12214149

[5] Bezemer ID, Doggen CJM, Vos HL, Rosendaal FR. No association between the common MTHFR 677C->T polymorphism and venous thrombosis: results from the MEGA study. Arch Intern Med [Internet]. 2007;167:497–501. Available from: http://www.ncbi.nlm.nih.gov/pubmed/17353498 10.1001/archinte.167.5.497 17353498

[6] Næss IA, Christiansen SC, Romundstad PR, Cannegieter SC, Blom HJ, Rosendaal FR et al. Prospective study of homocysteine and MTHFR 677TT genotype and risk for venous thrombosis in a general population– results from the HUNT 2 study. Br J Haematol [Internet]. 2008;141:529–35. Available from: 10.1111/j.1365-2141.2008.07073.x 10.1111/j.1365-2141.2008.07073.x 18318759

[7] Moll S, Varga EA, Homocysteine MTHFR, mutations. Circulation [Internet]. 2015;132:e6-9. Available from: http://www.ncbi.nlm.nih.gov/pubmed/26149435 10.1161/CIRCULATIONAHA.114.013311 26149435

[8] Hickey SE, Curry CJ, Toriello HV. ACMG practice guideline: lack of evidence for MTHFR polymorphism testing. Genet Sci. 2013;15:153–6. 10.1038/gim.2012.165 23288205

[9] Ghouse J, Tragante V, Ahlberg G, Rand SA, Jespersen JB, Leinøe EB, et al. Genome-wide meta-analysis identifies 93 risk loci and enables risk prediction equivalent to Monogenic forms of venous thromboembolism. Nat Genet. 2023;55:399–409. 36658437 10.1038/s41588-022-01286-7

[10] Mishra A, Malik R, Hachiya T, Jürgenson T, Namba S, Posner DC, et al. Stroke genetics informs drug discovery and risk prediction across ancestries. Nature [Internet]. 2022;611:115–23. Available from: http://www.ncbi.nlm.nih.gov/pubmed/36180795 10.1038/s41586-022-05165-3 PMC9524349 36180795

[11] Adams HP, Bendixen BH, Kappelle LJ, Biller J, Love BB, Gordon DL et al. Classification of subtype of acute ischemic stroke. Definitions for use in a multicenter clinical trial. TOAST. Trial of Org 10172 in Acute Stroke Treatment. Stroke [Internet]. 1993;24:35–41. Available from: 10.1161/01.STR.24.1.35 10.1161/01.str.24.1.35 7678184

[12] Kanehisa M, Goto S. KEGG: kyoto encyclopedia of genes and genomes. Nucleic Acids Res [Internet]. 2000;28:27–30. Available from: http://www.ncbi.nlm.nih.gov/pubmed/10592173 10.1093/nar/28.1.27 PMC102409 10592173

[13] Eldjarn GH, Ferkingstad E, Lund SH, Helgason H, Magnusson OT, Gunnarsdottir K, et al. Large-scale plasma proteomics comparisons through genetics and disease associations. Nature. 2023;622:348–58. 37794188 10.1038/s41586-023-06563-x PMC10567571

[14] Ferkingstad E, Sulem P, Atlason BA, Sveinbjornsson G, Magnusson MI, Styrmisdottir EL, et al. Large-scale integration of the plasma proteome with genetics and disease. Nat Genet. 2021;53:1712–21. 34857953 10.1038/s41588-021-00978-w

[15] Loh PR, Tucker G, Bulik-Sullivan BK, Vilhjálmsson BJ, Finucane HK, Salem RM, et al. Efficient bayesian mixed-model analysis increases association power in large cohorts. Nat Genet. 2015;47:284–90. 25642633 10.1038/ng.3190 PMC4342297

[16] Connors JM. Thrombophilia testing and venous thrombosis. N Engl J Med. 2017;377:1177–87. 28930509 10.1056/NEJMra1700365

[17] Yamada K, Chen Z, Rozen R, Matthews RG. Effects of common polymorphisms on the properties of recombinant human methylenetetrahydrofolate reductase. Proc Natl Acad Sci USA [Internet]. 2001;98:14853–8. Available from: http://www.ncbi.nlm.nih.gov/pubmed/11742092 10.1073/pnas.261469998 PMC64948 11742092

[18] Friedman G, Goldschmidt N, Friedlander Y, Ben-Yehuda A, Selhub J, Babaey S, et al. A common mutation A1298C in human methylenetetrahydrofolate reductase gene: association with plasma total homocysteine and folate concentrations. J Nutr. 1999;129:1656–61. 10460200 10.1093/jn/129.9.1656

[19] Deloughery TG, Hunt BJ, Barnes GD, Connors JM, Ay C, Barco S et al. A call to action: MTHFR polymorphisms should not be a part of inherited thrombophilia testing. Res Pract Thromb Haemost [Internet]. 2022;6:e12739. Available from: https://linkinghub.elsevier.com/retrieve/pii/S2475037922012353 10.1002/rth2.12739 PMC9175241 35702587

[20] White RH, Garcia M, Sadeghi B, Tancredi DJ, Zrelak P, Cuny J, et al. Evaluation of the predictive value of ICD-9-CM coded administrative data for venous thromboembolism in the United States. Thromb Res. 2010;126:61–7. 20430419 10.1016/j.thromres.2010.03.009

